# Bisphenols exert detrimental effects on neuronal signaling in mature vertebrate brains

**DOI:** 10.1038/s42003-021-01966-w

**Published:** 2021-04-12

**Authors:** Elisabeth Schirmer, Stefan Schuster, Peter Machnik

**Affiliations:** Department of Animal Physiology, University of Bayreuth, Bayreuth, Germany

**Keywords:** Toxicology, Neurophysiology, Risk factors

## Abstract

Bisphenols are important plasticizers currently in use and are released at rates of hundreds of tons each year into the biosphere^1–3^. However, for any bisphenol it is completely unknown if and how it affects the intact adult brain^4–6^, whose powerful homeostatic mechanisms could potentially compensate any effects bisphenols might have on isolated neurons. Here we analyzed the effects of one month of exposition to BPA or BPS on an identified neuron in the vertebrate brain, using intracellular in vivo recordings in the uniquely suited Mauthner neuron in goldfish. Our findings demonstrate an alarming and uncompensated in vivo impact of both BPA and BPS—at environmentally relevant concentrations—on essential communication functions of neurons in mature vertebrate brains and call for the rapid development of alternative plasticizers. The speed and resolution of the assay we present here could thereby be instrumental to accelerate the early testing phase of next-generation plasticizers.

## Abstract (web summary)

Elisabeth Schirmer, Stefan Schuster and Peter Machnik investigated the effects of bisphenols A and S on neuronal functioning. Using in vivo recordings in goldfish they demonstrate that basic neuronal properties such as action potentials and synaptic transmission are perturbed after chronic exposure to bisphenols.

## Introduction

Plasticizers are essential ingredients to plastic production^7,8^. However, upon degradation of plastic products these additives are released into the environment in large quantity, making plasticizer contamination a serious environmental issue and potential risk for our health^1,9–12^. For example, 8 million tons of the plasticizer bisphenol A (BPA; 2,2-bis-(4-hydroxyphenyl)-propane; CAS Registry No. 80-05-7) are produced worldwide each year and 100 tons per year are released into the biosphere^2,3,13,14^, making BPA ubiquitously present in the environment from surface water to breast milk^1^. Initially considered harmless, its various effects on hormonal balance, reproduction, and development in vertebrates^6,10,12,15,16^ have led to its replacement—particularly in baby products—by other bisphenols, most notably bisphenol S (BPS; 4,4’-sulfonyldiphenol; CAS Registry No. 80-09-1)^4,5^, which is presently available in the EU at rates of 10,000 tons per year^17^. Evidence, however, is mounting that also BPS might not be unproblematic^1,4,18–24^. Its almost 100-fold higher solubility in water compared to BPA makes BPS now readily detectable in aqueous environments^25,26^. Studies in fish models, however, indicate that exposition not only to BPA, but also to BPS results in developmental deformities, impaired and abnormal behavior^27–30^.

Here we demonstrate an alarming effect of bisphenols that has, to our knowledge, never been described before: we describe here clear and alarming effects of both BPA and its substitute BPS on neuronal functionality in the mature vertebrate brain despite the powerful homeostatic mechanisms that act in vivo^31,32^ and that could in principle compensate for any effects seen in vitro^33,34^. Our findings make it very likely that bisphenols also affect the adult human brain and can, among other aspects, change the delicate balance between excitation and inhibition, which is seen as the basis of several neuronal disorders^6,35,36^. Our findings call for new approaches to speed up the development and efficient pre-testing of alternative plasticizers. Specifically, the assay that we describe here can rapidly and accurately provide comprehensive information on effects on the mature brain and should therefore be part of a battery of efficient tests in the development of future plasticizers.

## Results

### Assaying neural function in vivo

**Fig. 1:**
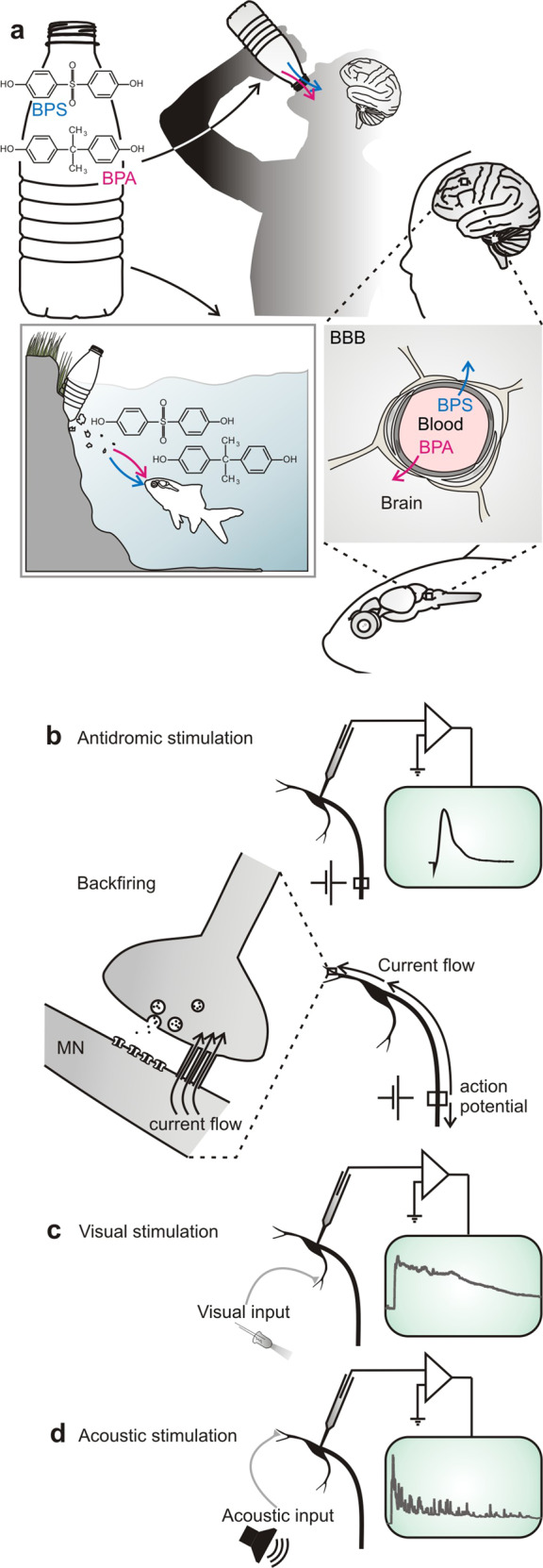
Studying the in vivo effect of bisphenols on neural functionality in the mature CNS of a vertebrate. **a** Bisphenols are additives in plastic products. As plastic degrades these substances are released into the environment and are then able to pass the blood–brain barrier (BBB). Bisphenol A (BPA), the most widely used plasticizer, is released at millions of tons per year and has devastating effects, for instance during ontogeny. Its substitute bisphenol S (BPS) also appears problematic, but for both bisphenols any effect on the mature brain is unknown. **b**–**d** In vivo intracellular recordings in the central nervous system using the identified Mauthner neuron (MN) of the goldfish provide a comprehensive and rapid assay of fundamental aspects of the action of bisphenols in the intact adult brain: The effect on ion channels, chemical and electric synaptic transmission can be studied by antidromically stimulating the axon and monitoring backfiring by currents that spread to the presynaptic sites (**b**). The effect on visual (**c**) and acoustic (**d**) processing as well as transmission and integration at the dendrite can be studied by recording postsynaptic potentials after sensory stimulation.

To study the effect of exposure to bisphenols on the adult vertebrate brain (Fig. 1a), the Mauthner neuron of fish and some amphibians is an ideal substrate. It is one of the very few neurons in the vertebrate CNS that can be identified individually from one animal to the next and that is readily accessible to intracellular in vivo recording^37^. Therefore, it has been a major source of insight into fundamental mechanisms of synaptic communication in the vertebrate CNS^38^. The two Mauthner neurons are essential for triggering the vital escape in response to suddenly approaching predators^39,40^. This requires the Mauthner neuron to integrate information from all sensory systems. Hence, intracellular recordings from the Mauthner neuron can rapidly and highly sensitively assay a number of key aspects of neuronal and circuit function. In our tests, we elicited action potentials in the Mauthner neurons antidromically (Fig. 1b), i.e., by stimulating their large axons (see “Methods”), and quantitatively analyzed several of its characteristics. Additionally, we presented sensory stimuli to activate visual (Fig. 1c) and acoustic (Fig. 1d) processing and recorded postsynaptic potentials (PSPs) to analyze the integration of sensory information in the Mauthner neuron.

### Effects on the action potential

**Fig. 2:**
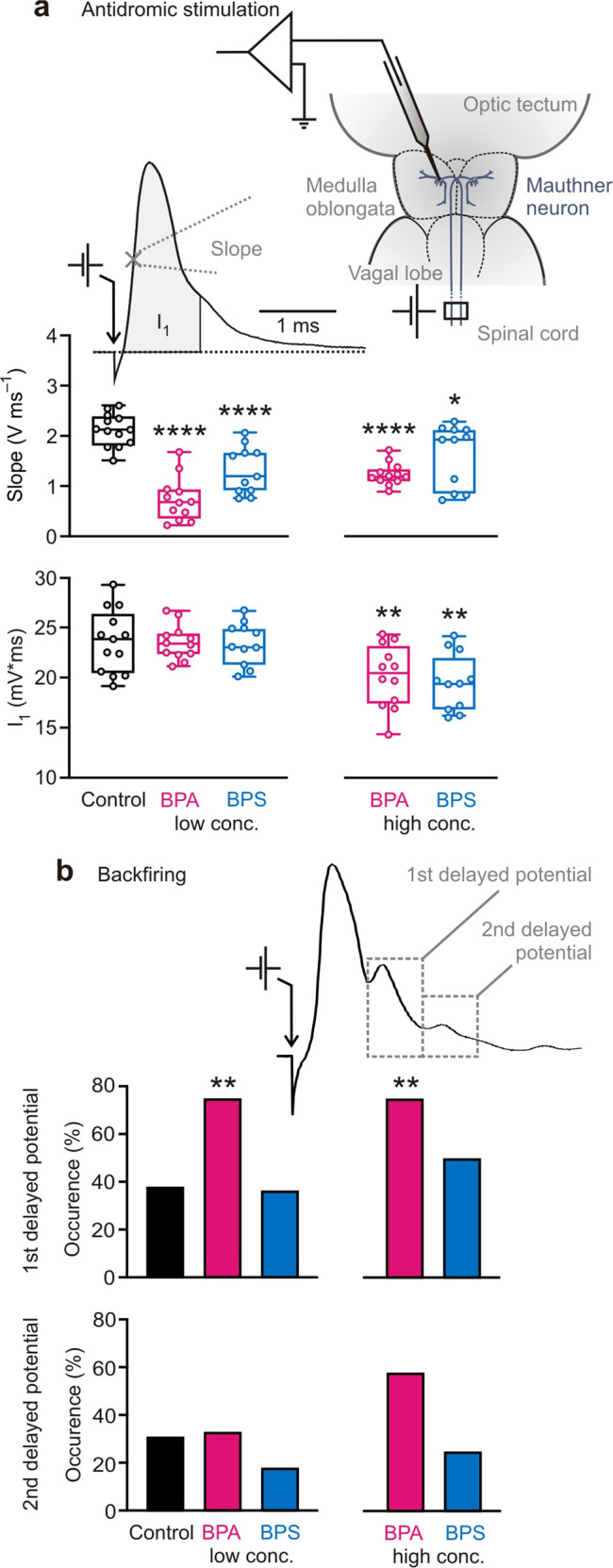
BPA and BPS both affect central neurons. **a** BPA and BPS both affected the action potential in the Mauthner neuron. In high and low concentration, they significantly decreased the slope of the action potential. In high concentration, they additionally reduced the area I_1_. The sketch indicates the experimental setting for antidromic stimulation and intracellular recording from the Mauthner soma. The exemplary action potential from a control fish illustrates the interval in which slope and I_1_ were determined. **b** Changes in the occurrence of delayed potentials (DPs) due to backfiring (see Fig. 1b) after bisphenol exposition indicate an impact on synaptic transmission in the CNS. BPA (but not BPS) increased the occurrence of DPs in exposed fish. Low conc. = 10 µg L^−1^; high conc. = 1 mg L^−1^; *N*_(Control)_ = 13 independent samples; *N*_(10 µg L_^−1^_BPA)_ = 12 independent samples; *N*_(1 mg L_^−1^_BPA)_ = 12 independent samples; *N*_(10 µg L_^−1^_BPS)_ = 11 independent samples; *N*_(1 mg L_^−1^_BPS)_ = 11 independent samples; differently treated groups are indicated by color; whiskers show the minimum and the maximum value, respectively; significant differences between groups and control are indicated by asterisk(s); * indicates *P* < 0.05; ** indicates *P* ≤ 0.01; **** indicates *P* ≤ 0.0001.

We discovered that even at environmentally relevant concentration of 10 µg L^−1^ one month of exposure to BPA or BPS massively reduced the maximal initial slope of the action potential (Fig. 2a) in vivo. In the controls (exposed to the solvent DMSO) the maximal initial slope was 2.13 ± 0.33 V ms^−1^ (*N* = 13 independent animal samples; *n* = 9 to 31 measurements per fish). 10 µg L^−1^ BPA reduced it to 0.68 ± 0.44 V ms^−1^ (*N* = 12 independent animal samples; 76 ≤ *n* ≤ 114; one-way ANOVA: *F* = 17.55; *R*^*2*^ = 0.5899; *P* < 0.0001; Dunnett test: mean diff.: 1.39; confidence interval of diff.: 0.95–1.82; *P* < 0.0001) and 1 mg L^−1^ to 1.19 ± 0.22 V ms^−1^ (*N* = 12 independent animal samples; 17 ≤ *n* ≤ 19; Dunnett test: mean diff.: 0.89; confidence interval of diff.: 0.46–1.33; *P* < 0.0001). Exposure to 10 µg L^−1^ BPS reduced the maximal initial slope of the action potential to 1.20 ± 0.47 V ms^−1^ (*N* = 11 independent animal samples; 86 ≤ *n* ≤ 104; Dunnett test: mean diff.: 0.80; confidence interval of diff.: 0.35–1.24; *P* < 0.0001) and 1 mg L^−1^ to 1.93 ± 0.62 V ms^−1^ (*N* = 11 independent animal samples; 20 ≤ *n* ≤ 159; Dunnett test: mean diff.: 0.48; confidence interval of diff.: 0.03–0.92; *P* = 0.032). Because we also noticed effects on the time course of the action potential, we analyzed the time-integrated action potential, taking the area I_1_ for the first ms after onset (see Fig. 2a). Interestingly, here only the higher plasticizer concentration showed an effect: In controls, I_1_ was 23.8 ± 3.1 mV ms (*N* = 13 independent animal samples; 9 ≤ *n* ≤ 31). 1 mg L^−1^ BPA reduced the integrated action potential in the first ms of its duration to 20.5 ± 3.1 mV ms (*N* = 12 independent animal samples; 17 ≤ *n* ≤ 19; one-way ANOVA: *F* = 6.249; *R*^*2*^ = 0.3387; *P* < 0.0001; Dunnett test: mean diff.: 3.45; confidence interval of diff.: 0.82–6.08; *P* = 0.0055). With 1 mg L^−1^ BPS, I_1_ was 19.4 ± 2.9 mV ms (*N* = 11 independent animal samples; 20 ≤ *n* ≤ 159; Dunnett test: mean diff.: 4.09; confidence interval of diff.: 1.40–6.78; *P* = 0.001).

### BPA increases neuronal backfiring

The action potential of the Mauthner neuron can backfire to presynaptic sites through electrical synapses (Fig. 1b). These are part of the mixed “club-ending” synapses that convey acoustic input onto the lateral dendrite of the Mauthner neuron. The resulting depolarization of the presynaptic site can then again cause transmitter release, giving rise to a delayed potential (DP) that lags the action potential by about 1 ms^41^. If this also backfires, even a second DP can be generated. The DPs are therefore a valuable tool for assessing how bisphenols affect electrical synapses and presynaptic transmitter release. Figure 2b illustrates two exemplary DPs. They typically followed 0.86 ± 0.06 ms (amplitude 10.3 ± 3.8 mV; *N* = 5 independent animal samples; *n* = 9 to 31 measurements per fish; first DP) and 1.44 ± 0.05 ms (amplitude 4.9 ± 1.9 mV; *N* = 4 independent animal samples; *n* = 9–31; second DP) after onset of the action potential (Supplementary Fig. 1b). In the control group a pair of DPs occurred consistently in 31% (4 of 13) of the fish, a single DP in 38% (5 of 13). Consistent with the notion that the 2nd DP is caused by transmitter release due to the presynaptic spreading of the 1st DP, we found that the amplitude of the 2nd DP correlates with the amplitude of the first (Supplementary Fig.1c; *N* = 20 independent animal samples; 9 ≤ *n* ≤ 114; Spearman correlation: *P* = 0.01) and was absent when the first one was absent. In contrast, we found no correlation between the amplitude of the 1st DP and the amplitude of the action potential (Supplementary Fig.1c; *N* = 33 independent animal samples; 9 ≤ *n* ≤ 114; Spearman correlation: *P* = 0.45). Strikingly, one month of exposition to BPA strongly affected backfiring in vivo through mixed electrical and chemical synapses. While both BPA and BPS did not affect the amplitudes of the DPs (Supplementary Fig.1d; one-way ANOVA: *F* ≤ 1.539; *R*^*2*^ ≤ 0.1751; *P* ≥ 0.217), specifically BPA (but not BPS) dramatically increased the occurrence of DPs: In the group of fish exposed to 1 mg L^−1^ BPA as well as in that exposed to 10 µg L^−1^ BPA the first delayed potential occurred in 75% (9 of 12) (Wilcoxon test for difference from control: *P* = 0.009). An additional second DP occurred in 58% (7 of 12) of fish exposed to 1 mg L^−1^ BPA (Wilcoxon test for difference from control: *P* = 0.086) and in 33% (4 of 12) of fish exposed to 10 µg L^−1^ BPA (Wilcoxon test for difference from control: *P* = 0.13). BPA thus strongly increased neuronal backfiring. In light of the findings below, this is a likely consequence of increased transmission at the glutamatergic mixed synapses and increased spreading of the action potential to presynaptic sites.

### Bisphenols affect acoustic processing

**Fig. 3:**
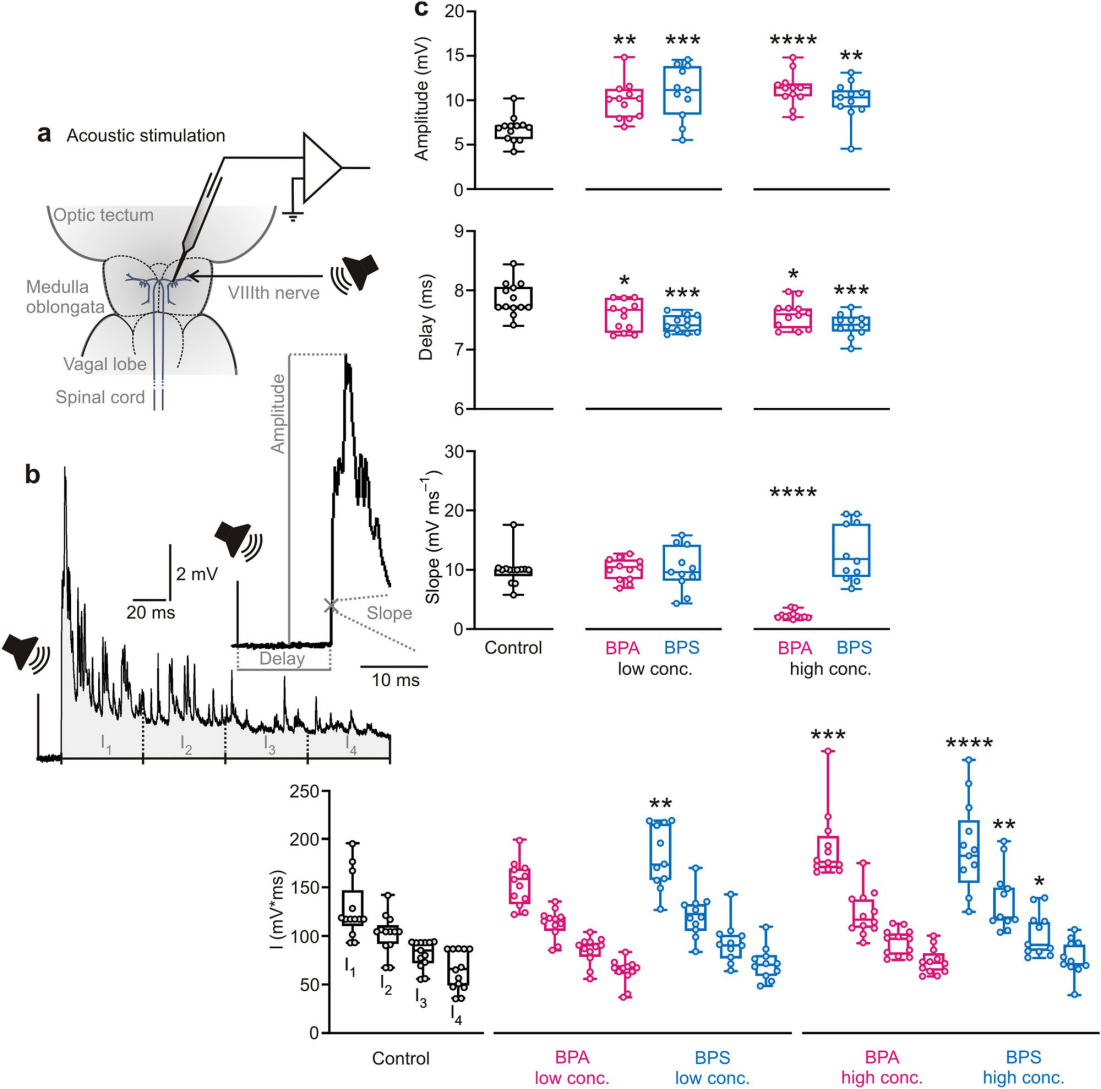
Both BPA and BPS affect auditory processing. **a** Sketch of experimental setting for acoustic stimulation and **b** an exemplary acoustically induced postsynaptic potential (PSP) from a control fish with illustration of how measurements were taken. **c** Both concentrations of BPA increased the PSP amplitude and decreased delay of the PSP relative to stimulus onset. Additionally, the high BPA concentration increased area I_1_ and decreased the slope of the PSP. BPS exposition also increased the amplitude of the acoustically induced PSP and I_1_. The high BPS concentration also increased the areas I_2_ and I_3_. Additionally, exposition to BPS shortened the delay. Low conc. = 10 µg L^−1^; high conc. = 1 mg L^−1^; *N*_(Control)_ = 13 independent samples; *N*_(10 µg L_^−1^_BPA)_ = 12 independent samples; *N*_(1 mg L_^−1^_BPA)_ = 12 independent samples; *N*_(10 µg L_^−1^_BPS)_ = 11 independent samples; *N*_(1 mg L_^−1^_BPS)_ = 11 independent samples; differently treated groups are indicated by color; whiskers show the minimum and the maximum value, respectively; significant differences between groups and control are indicated by asterisk(s); * indicates *P* < 0.05; ** indicates *P* ≤ 0.01; *** indicates *P* ≤ 0.001; **** indicates *P* ≤ 0.0001.

One month of exposure to BPA or BPS had striking and uncompensated effects on the PSPs that were elicited by our broadband acoustic pulse. The experimental setting and an exemplary PSP of a control animal are shown in Fig. 3a, b. Strikingly, the bisphenols affected basically all aspects of the acoustic PSP. BPA and BPS both increased the amplitude, the temporal integral and its longtime decay. Maximum amplitude of the PSPs was increased from 7.1 ± 1.4 mV (*N* = 13 independent animal samples; between *n* = 8–29 measurements per fish) in the controls to 11.2 ± 3.0 mV (*N* = 11 independent animal samples; 16 ≤ *n* ≤ 49) with 10 µg L^−1^ BPS and to 10.4 ± 2.3 mV (*N* = 11 independent animal samples; 9 ≤ *n* ≤ 46) with 1 mg L^−1^. 10 µg L^−1^ BPA increased the maximum amplitude to 10.2 ± 2.1 mV (*N* = 12 independent animal samples; 10 ≤ *n* ≤ 52) and 1 mg L^−1^ to 11.4 ± 1.9 mV (*N* = 12 independent animal samples; 11 ≤ *n* ≤ 23; Fig. 3c; one-way ANOVA: *F* = 6.994; *R*^*2*^ = 0.3569; *P* < 0.0001; Dunnett test: *P* ≤ 0.0032). To assay the temporal concentration of the changes in membrane potential, we considered the temporal integral in four consecutive intervals, 50 ms each (Fig. 3b; integrals I_1_ to I_4_). This analysis showed a clear increase of the first integral (Fig. 3c; one-way ANOVA: *F* = 6.479; *R*^*2*^ = 0.3396; *P* < 0.0001) from 117.5 ± 32.1 mV ms (*N* = 13 independent animal samples; 8 ≤ *n* ≤ 29) in the controls to 173.6 ± 32.6 mV ms (*N* = 11 independent animal samples; 16 ≤ *n* ≤ 49) with 10 µg L^−1^ BPS (Dunnett test: mean diff.: −53.74; confidence interval of diff.: −91.16 to −16.32; *P* = 0.002) and to 188.9 ± 47.2 mV ms (*N* = 11 independent animal samples; 9 ≤ *n* ≤ 46) with 1 mg L^−1^ BPS (Dunnett test: mean diff.: −67.47; confidence interval of diff.: −104.9 to −30.05; *P* < 0.0001). BPA significantly increased I_1_ in high (Dunnett test: mean diff.: −63.64; confidence interval of diff.: −100.20 to −27.07; *P* = 0.0001), but not in low concentration (Dunnett test: mean diff.: −25.96; confidence interval of diff.: −62.53 to 10.61; *P* = 0.2516). In fish exposed to 1 mg L^−1^ BPA, I_1_ was 176.4 ± 35.8 mV ms (*N* = 12 independent animal samples; 11 ≤ *n* ≤ 23). For the acoustic PSPs caused by our stimulus, the time integrals decayed exponentially (*y* = *y*_0_ * exp (−*k* * *x*)) (goodness of fit: *r*^2^ ≥ 0.54), but with larger rate constants in bisphenol-exposed fish (*k*_control_ = 0.22; *k*_BPS_ ≥ 0.31; *k*_BPA_ ≥ 0.30). The increase of rate of decay from I_1_ to I_4_ thereby was significant in fish exposed to 1 mg L^−1^ BPA (Fig. 3c; one-way ANOVA: *F* = 3.357; *R*^*2*^ = 0.1991; *P* = 0.0159; Dunnett test: mean diff.: 0.117; confidence interval of diff.: 0.030 to 0.203; *P* = 0.0047) and those exposed to 10 µg L^−1^ BPS (Dunnett test: mean diff.: 0.095; confidence interval of diff.: 0.004–0.186; *P* = 0.0371). BPA and BPS significantly reduced the delay of the PSP relative to stimulus onset (Fig. 3c; one-way ANOVA: *F* = 6.426; *R*^*2*^ = 0.3377; *P* < 0.0001; Dunnett test: *P* ≤ 0.0399). Delay was 7.71 ± 0.28 ms (*N* = 13 independent animal samples; 8 ≤ *n* ≤ 29) in controls. 10 µg L^−1^ BPA reduced it to 7.62 ± 0.26 ms (*N* = 12 independent animal samples; 10 ≤ *n* ≤ 52) and 1 mg L^−1^ BPA to 7.60 ± 0.23 ms (*N* = 12 independent animal samples; 11 ≤ *n* ≤ 23). 10 µg L^−1^ BPS reduced the delay to 7.41 ± 0.14 ms (*N* = 11 independent animal samples; 16 ≤ *n* ≤ 49) and 1 mg L^−1^ BPS to 7.42 ± 0.19 ms (*N* = 11 independent animal samples; 9 ≤ *n* ≤ 46). Finally, 1 mg L^−1^ BPA significantly reduced the maximal initial slope of the PSP from 10.0 ± 2.7 mV ms^−1^ (*N* = 13 independent animal samples; 8 ≤ *n* ≤ 29) in the control group to 2.0 ± 0.7 mV ms^−1^ (*N* = 12 independent animal samples; 11 ≤ *n* ≤ 23) (one-way ANOVA: *F* = 16.62; *R*^*2*^ = 0.5689; *P* < 0.0001; Dunnett test: mean diff.: 7.72; confidence interval of diff.: 4.55–10.90; *P* < 0.0001).

In conclusion, both bisphenols had striking effects on almost all functionally relevant aspects of the acoustic PSP. Most remarkably they increased the efficiency at which the acoustic stimulus excited the Mauthner neuron. Because at least BPS is thought to negatively affect sensory hair cells^42^, a decrease rather than an increase of the amplitude of acoustic PSPs would have been expected. Our findings, therefore, suggest important and unbalanced excitatory effects of BPA and BPS on (glutamatergic^41^) synaptic transmission in the CNS.

### Bisphenols affect visual processing

**Fig. 4:**
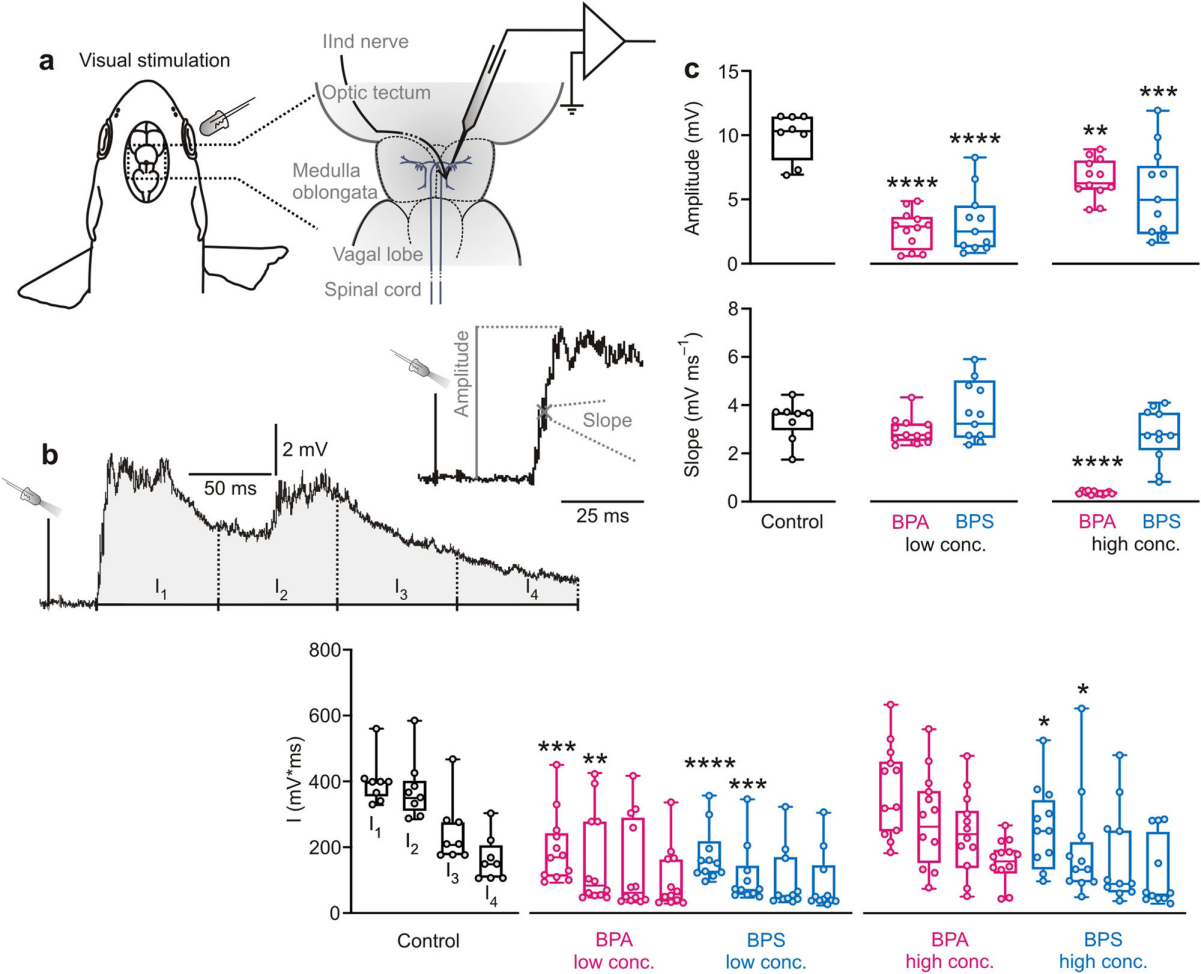
Both BPA and BPS affect visual processing. **a** Sketch of experimental setting for visual stimulation to emphasize efficiency of the system: only stimulation needs to be changed, but recording is kept (cf. Figs. 2a and 3a). **b** shows an exemplary visually induced PSP from a control fish and how measurements were taken. **c** Both BPA and BPS affected the visually induced PSP. BPA and BPS significantly reduced its amplitude. BPA in high concentration (but not BPS) additionally reduced PSP slope. In contrast, after exposition to BPS and to low BPA concentration the beginning of the PSP (first 150 ms; I_1_ and I_2_) was reduced in area. Low conc. = 10 µg L^−1^; high conc. = 1 mg L^−1^; *N*_(Control)_ = 8 independent samples; *N*_(10 µg L_^−1^_BPA)_ = 12 independent samples; *N*_(1 mg L_^−1^_BPA)_ = 12 independent samples; *N*_(10 µg  L_^−1^_BPS)_ = 11 independent samples; *N*_(1 mg L_^−1^_BPS)_ = 11 independent samples; differently treated groups are indicated by color; whiskers show the minimum and the maximum value, respectively; significant differences between groups and control are indicated by asterisk(s); * indicates *P* < 0.05; ** indicates *P* ≤ 0.01; *** indicates *P* ≤ 0.001; **** indicates *P* ≤ 0.0001.

The bisphenols not only affected acoustic circuits but had striking effects on the visual PSP. The experimental setting and an exemplary PSP are shown in Fig. 4a, b. In contrast to their effect on the acoustic PSP, BPA and BPS strongly reduced the amplitude of the visual PSPs (Fig. 4c; one-way ANOVA: *F* = 17.83; *R*^*2*^ = 0.6058; *P* < 0.0001; Dunnett test: *P* ≤ 0.0046). In controls, PSP amplitude was 10.4 ± 1.8 mV (*N* = 8 independent animal samples; *n* = 7 to 21 measurements per fish). 10 µg L^−1^ BPA reduced it to 2.9 ± 1.5 mV (*N* = 12 independent animal samples; 8 ≤ *n* ≤ 27) and 1 mg L^−1^ BPA to 6.3 ± 1.5 mV (*N* = 12 independent animal samples; 10 ≤ *n* ≤ 21). In BPS exposed fish, PSP amplitude was 2.5 ± 2.4 mV (*N* = 11 independent animal samples; 7 ≤ *n* ≤ 31) for 10 µg L^−1^ and 5.0 ± 3.4 mV (*N* = 11 independent animal samples; 11 ≤ *n* ≤ 25) for 1 mg L^−1^ BPS. In addition, 1 mg L^−1^ BPA (but not the low concentration of BPA tested or BPS) also drastically reduced the maximal initial slope of the PSPs from 3.66 ± 0.82 mV ms^−1^ (*N* = 8 independent animal samples; 7 ≤ *n* ≤ 21) to only 0.32 ± 0.07 mV ms^−1^ (*N* = 12 independent animal samples; 10 ≤ *n* ≤ 21; one-way ANOVA: *F* = 24.51; *R*^*2*^ = 0.6788; *P* < 0.0001; Dunnett test: mean diff.: 3.00; confidence interval of diff.: 2.04 to 3.96; *P* < 0.0001). BPA and BPS additionally affected the temporal integral of the PSPs (Fig. 4c; one-way ANOVA: *F* = 11.43; *R*^*2*^ = 0.4964; *P* < 0.0001) with the first integral (of 75 ms duration) strongly decreased from 398.0 ± 71.5 mV ms (*N* = 8 independent animal samples; 7 ≤ *n* ≤ 21) in the control group to 169.5 ± 107.8 mV ms with 10 µg L^−1^ BPA (*N* = 12 independent animal samples; 8 ≤ *n* ≤ 27; Dunnett test: mean diff.: 205.4; confidence interval of diff.: 83.8–327.1; *P* = 0.0003), to 150.9 ± 82.8 mV ms with 10 µg L^−1^ BPS (*N* = 11 independent animal samples; 7 ≤ *n* ≤ 31; Dunnett test: mean diff.: 227.1; confidence interval of diff.: 103.3–350.9; *P* < 0.0001) and to 250.0 ± 125.4 mV ms with 1 mg L^−1^ BPS (*N* = 11 independent animal samples; 11 ≤ *n* ≤ 25; Dunnett test: mean diff.: 134.3; confidence interval of diff.: 10.5–258.2; *P* = 0.0293).

### Effects of EE2

For many of the varied non-neuronal effects of bisphenols their structural similarity with estrogens is crucial^15,16,22^. A series of experiments was therefore aimed at exploring whether this might also apply, to some extent, to the strong neuronal effects we describe here. We therefore ran experiments just as with BPA and BPS (Figs. 2–4) and also with one month of exposure, but with fish exposed not to any bisphenols but to ethinyl estradiol (EE2) at a concentration of 1 mg L^−1^. A full account of all results obtained in these experiments is given in Supplementary Table 1. Table 1 highlights all significant effects that we were able to detect with EE2 and compares their occurrence and direction with those we found after BPA and BPS exposition (at any concentration). EE2 highly significantly increased action potential amplitude and shortened the delay, after which an action potential followed after spinal cord stimulation, effects that we found neither with BPA nor BPS at any concentration. However, all other effects, including their direction, were strikingly similar as with the bisphenols. This might suggest that at least some of the neuronal effects of the bisphenols could also result from their similarity with estrogens.

**Table 1:** The spectrum of significant effects on neuronal function found after one month of exposition to either ethinyl estradiol (EE2) at 1 mg L^−1^ or bisphenols (BPA or BPS) at any of the concentrations we examined (10 µg L^−1^ and 1 mg L^−1^).

Substance	EE2	BPA	BPS
Effect on antidromically induced action potential			
Amplitude	↑		
Delay	↓		
Slope	↓	↓	↓
Area		↓	↓
1st DPs	↑	↑	
Effect on auditory induced PSPs			
Amplitude	↑	↑	↑
Delay		↓	↓
Slope		↓	
Area	↑	↑	↑
Effect on visually induced PSPs			
Amplitude	↓	↓	↓
Delay			
Slope		↓	
Area	↓	↓	↓

Based on data shown in Figs. 2–4 and Supplementary Table 1.

↓Indicate a significant decrease in comparison to control and ↑ a significant increase; free fields represent values that have not changed significantly in comparison to control.

### Acute effects of BPA and BPS

One month of exposition to BPA or BPS at concentrations of 10 µg L^−1^ or 1 mg L^−1^ caused strong effects on all aspects of neuronal function. Our final series of experiments was therefore aimed at testing whether the effects required prolonged exposition or might at least partly be seen in acute experiments. In these, the tests shown in Figs. 2–4 were run for a total of 20 min in untreated fish, to establish baseline properties. Then either BPS (*N* = 6 independent animal samples) or BPA (*N* = 7 independent animal samples) was added so that the fish now faced a concentration of 10 µg L^−1^. After 10 min of incubation the 20 min stimulus program was run again. Subsequently, the concentration of the respective bisphenol was increased to 1 mg L^−1^ and an incubation of 10 min was allowed before the stimulus program was given. At the measurements at the higher concentration, the fish had been exposed to bisphenol for a comparably brief time between 40 min (10 min at the high concentration plus 30 min at the lower concentration) and 60 min. The results of all three series (baseline, 10 µg L^−1^, 1 mg L^−1^) for both BPA and BPS are reported in detail in Supplementary Table 2. In none of the experiments did the acute exposition cause any significant deviations from baseline (RM one-way ANOVA: *F* ≤ 3.59; *R*^*2*^ ≤ 0.42; *P* ≥ 0.07). These findings therefore suggest that the strong neuronal effects seen after one month of exposure do not establish quickly after short exposure of only about 1 h.

## Discussion

Our in vivo recordings demonstrate strikingly strong and uncompensated effects of bisphenols on all aspects of neuronal function in the adult vertebrate brain, from the action potential, the balance between excitatory and inhibitory inputs to auditory and visual sensory circuits. Our findings have been obtained in a particularly accessible identified neuron in the mature vertebrate CNS, the Mauthner neuron of the goldfish. This neuron is particularly interesting for an analysis of whether the effects of bisphenols could be buffered: Buffering should be particularly strong in this neuron, because its inputs and outputs are essential for driving life-saving escapes. Although the effects of bisphenols certainly vary between individual neurons and across species, our findings clearly establish that the effects of bisphenols on the nervous system are by no means restricted to developing brains. Rather, being exposed to either BPA or BPS at the environmentally relevant concentration^2,43^ of 10 µg L^−1^ for one month strongly affects neuronal function in the adult brain.

On the more optimistic side, our findings demonstrate that it is possible to quickly gain sensitive information on basic neuronal functions—from generation of the action potential, synaptic transmission to auditory and visual function—by using multisensory integration in identified neurons such as the Mauthner neuron as a powerful tool. Studying the postsynaptic potentials in response to acoustic or visual stimulation showed clear effects of both bisphenols on sensory systems and on central processing. Although it has been suggested that BPA damages sensory hair cells in fish and amphibia^42^, we find that BPA—surprisingly—increased the amplitude of acoustical PSPs and that BPS acted similarly. These effects could be explained by a strong effect of both BPA and BPS on excitatory synaptic transmission. However, our findings also demonstrate that not all synapses are equally potentiated: For instance, backfiring through the mixed synapses was strongly increased by BPA, but not affected by BPS. Furthermore, the visual PSPs were clearly reduced both after exposition to BPA or BPS, which would only for BPS be attributable to an effect on retinal function^44^.

The strong effects we find here and the apparent lack of efficient buffering are alarming. The effects of bisphenols have previously been discussed mainly from a developmental point of view (causing the ban of BPA from baby products in some countries) or from its varied endocrinological effects. Now we face an additional danger whose effect on healthy humans and on patients with neurological deficits is difficult to foresee. Offsetting balances in brains is the basis of severe neurological disorders^6,24,27,30,35,36^ and so our findings must be taken very seriously. What is most needed, is an effort to develop a new generation of plasticizers combined with an efficient but sufficiently broad and sensitive array of tests to quickly detect and sort out substances that bear large environmental and health risks^5,11^. The tests we described here are particularly efficient and can quickly assay effects on neuronal functions. Together with similarly sensitive assays they could guide our way to the urgently needed next-generation plasticizers.

## Methods

### Animals and treatment

We used *N* = 98 goldfish (*Carassius auratus*, Cypriniformes) of either sex with an average standard length of 69.5 ± 7.8 mm (range from 56.5 to 100 mm) and an average body weight of 10.3 ± 3.8 g (range from 6.7 to 20.8 g). The fish were obtained from an authorized specialist retailer (Aquarium Glaser GmbH, Rodgau, Germany). Prior start of the project, fish were kept for at least 4 weeks in large glass tanks (250 × 50 × 50 (cm)) filled with fresh water (water conductivity: 300 µS cm^−1^; pH 7.5; total hardness of water: 7.7°dH; NH_4_^+^ < 10 µg L^−1^; NO_2_^−^ < 5 µg L^−1^; NO_3_^−^ < 5 mg L^−1^) at a water temperature of 20 °C. Light/dark photoperiod was 12:12 h. Fish were fed once a day with common fish food (sera goldy; sera GmbH, Heinsberg, Germany). After this period of acclimatization and quarantine, fish were checked for disorders and for responsiveness to visual and acoustic stimuli. We only chose healthy and responsive fish for the experiments. They were divided randomly into experimental groups exposed either to bisphenol A (BPA; 4,4’-(propane-2,2-diyl)-diphenol), bisphenol S (BPS; 4,4’-sulfonyldiphenol) or to ethinyl estradiol (EE2; 17α-ethinyl-1,3,5(10)-oestratrien-3,17β-diol). BPA and BPS were obtained in granular form from Sigma-Aldrich (Steinheim, Germany). EE2 was obtained in powder form from Merck KGaA (Darmstadt, Germany). For application, they were dissolved in dimethyl sulfoxide (DMSO), with a final DMSO concentration of 0.001% and added in the required concentration to the water.

Two experimental groups (7 fish each) were used to test for acute effects of BPA and BPS. Fish of these groups were not exposed to plasticizer prior to experiment. However, during Mauthner neuron intracellular recording, we added plasticizer (BPA or BPS) so that the fish acutely faced either BPA or BPS. Thereby, we were able to collect robust data for two concentrations (10 µg L^−1^ and 1 mg L^−1^) in *N* = 7 fish of the BPA group and *N* = 6 fish of the BPS group.

In six further groups (14 fish each), we tested for effects of BPA, BPS, and EE2 after a month of exposition. Fish of these groups were, respectively, exposed either to 10 µg L^−1^ BPA, 1 mg L^−1^ BPA, 10 µg L^−1^ BPS, 1 mg L^−1^ BPS, 1 mg L^−1^ EE2 or received only DMSO in the concentration used as dissolvent in the other groups. The latter group served as a control. By starting exposition at different times, experimental fish were exactly exposed to the respective chemical for 30 to 33 days. We were able to collect robust data in *N* = 13 fish of the control group, *N* = 12 fish of the 10 µg L^−1^ BPA group, *N* = 12 fish of the 1 mg L^−1^ BPA group, *N* = 11 fish of the 10 µg L^−1^ BPS group, *N* = 11 fish of the 1 mg L^−1^ BPS group and *N* = 10 fish of the 1 mg L^−1^ EE2 group. Two fish exposed to EE2 died prior experiment in the third week of exposition. Animal care procedures, surgical procedures, and experimental procedures were in accordance with all relevant guidelines and regulations of the German animal protection law and explicitly approved by state councils (Regierung von Unterfranken, Würzburg, Germany).

### Anesthesia and surgical procedure

Before starting surgery, the experimental fish was anaesthetized (2-phenoxyethanol in the concentration of 0.4 ml L^−1^) for 15 min in the water it was used to. Anesthesia was maintained also during surgery and during recording and the protocol is known not to affect neuronal functionality nor the acoustical or the visual system of goldfish^45^. To confirm the sufficiency of anesthetization, we carefully exerted pressure to the fish’s caudal peduncle after the fish had lost equilibrium, which normally would trigger vigorous escapes. Only when this stimulation (and subsequent handling) yielded no response, the fish was positioned in the recording chamber and given artificial respiration with aerated, anesthetic loaded water flowing via a tube through the fish’s mouth and out over the gills at a flow rate of 80 ml min^−1^. Here, we also used water of the same quality as for housing. Respiration water was delivered to the fish from a reservoir using a suitably adjusted pump (EHEIM universal 300; EHEIM GmbH & Co. KG, Deizisau, Germany; regular power: 300 L h^−1^, adjusted to 4.8 L h^−1^).

Access to the Mauthner neurons was achieved by using a bone rongeur to open the skull from above in the area of the hindbrain. To expose the medulla oblongata containing the pair of Mauthner neurons, the cerebellum was lifted up with a piece of filter paper and fixed in place. To stimulate the axons of the two Mauthner neurons, we additionally exposed a piece of the spinal column (about 5 mm in length) from the side in the region of the trunk (between 20 and 25 mm caudal from the position of the Mauthner somata) and confirmed suprathreshold stimulation of the Mauthner axons from the characteristic twitching of the experimental animal. To prepare for the intracellular in vivo recording the experimental animal was then immobilized by injecting d-tubocurarine (1 µg g^−1^ body weight; Sigma-Aldrich, Steinheim, Germany) in the core muscles. After finishing measurements, the experimental animal was sacrificed immediately and without recovery from anesthesia by mechanically destroying the brain. Finally, a necropsy was performed to check for any unnoticed diseases of inner organs. This confirmed that all fish of this study were healthy.

### Experimental procedure

For intracellular recordings, we used a bridge-mode amplifier (BA-01X; npi electronic GmbH, Tamm, Germany) in current clamp mode. Recording electrodes were pulled from 3 mm-glass capillaries (G-3; Narishige Scientific Instrument Lab, Tokyo, Japan) using a vertical electrode puller (PE-22; Narishige International Limited, London, UK). Filled with 5 M potassium acetate, they had a resistance between 4 and 7 MΩ. For moving and positioning the recording electrode, we used a motorized micromanipulator (MP-285; Sutter Instrument, Novato, CA, USA). We used established techniques to determine recording position from extracellular space and to ensure recordings are always taken in the soma of the Mauthner neuron^46^. The reference electrode was positioned in muscle tissue. Recordings were filtered (Hum Bug Noise Eliminator; Quest Scientific, North Vancouver, BC, Canada) and then digitized at the sample rate of 50 kHz using an A/D converter (Micro1401; Cambridge Electronic Design Limited, Cambridge, UK) and the acquisition software package Spike2 (version 6; Cambridge Electronic Design Limited, Cambridge, UK). For data analysis, we used custom-made software written in Python. After localization and identification of one of the two Mauthner neurons using well-established techniques^37,46^ and after establishing a stable intracellular recording of the Mauthner neuron, we applied a set of stereotyped stimuli to elicit Mauthner neuron responses. A set of stimuli, as designed for the present study, contained repeated antidromic activation of the Mauthner neuron and repeated acoustic and visual stimulation of the fish. Each of the stereotyped stimuli was consecutively presented to the fish at least 40 times per set. In total, presentation took about 20 min. To ensure stable intracellular recording, we continuously monitored the resting potential of the Mauthner neuron. In all cases, deviations were far less than our criterion (5%). We used electrical pulses (pulse duration: 10 µs; stimulation rate: 2 Hz) applied to the spinal cord to activate the Mauthner neuron antidromically. The electric pulses were delivered by a constant-voltage isolated stimulator (DS2A2 – Mk.II; Digitimer Ltd., Hertfordshire, UK). The desired pulse amplitude for antidromic stimulation was determined by first reducing amplitude until antidromic stimulation did not activate the Mauthner neuron anymore. Then amplitude was increased by 5 V above threshold. In the current study pulse amplitude for antidromic stimulation ranged from 15 to 40 V. Next, we tested the processing of sensory information in the Mauthner neuron. For acoustic stimulation, we used a multifunctional active loudspeaker (The box pro Achat 115 MA; Thomann GmbH, Burgebrach, Germany). The loudspeaker generated a short acoustical broadband pulse (duration: 1 ms; frequency distribution from 25 to 1000 Hz; peak amplitude at 300 Hz) with a sound pressure level (SPL) of 145 dB *re* 1 µPa. We measured SPL under water at the position of the fish in the recording chamber with a hydrophone (Type 8106; Brüel & Kjær, Nærum, Denmark). For visual stimulation, we used a light-emitting diode (LED; RS Components GmbH, Mörenfelden-Walldorf, Germany), which was positioned directly in front of the ipsilateral eye. The light flash used for visual stimulation had a duration of 7 ms. LED peak radiation at 569 nm was 700 µW m^−2^ nm^−1^ and the width at 100 µW m^−2^ nm^−1^ was 56 nm (range: 543–599 nm).

In experiments on the acute effect of plasticizers, each fish was given the set of stimuli three times. The first set of stimuli was presented 10 min after establishing intracellular Mauthner neuron recording and before adding any plasticizer and served to establish a baseline. Next, we added plasticizer (either BPA or BPS) to the water to reach a concentration of 10 µg L^−1^. After an incubation period of 10 min we recorded Mauthner neuron responses to our set of stimuli again. Then, we increased the concentration to 1 mg L^−1^, again gave 10 min for incubation before taking the final measurement. In total, all measurements were completed within 90 min of intracellular recording, and the maximum time any bisphenol could have acted in our acute experiments was 60 min. In fish exposed for a month to either BPA, BPS or EE2 we presented our set of stimuli 10 min after establishing intracellular Mauthner neuron recording only once. Per fish we needed 30 min of intracellular recording.

### Statistics and reproducibility

Statistical tests were run using the software package GraphPad Prism 8.2.1 (GraphPad Software, Inc., La Jolla, CA, USA) and performed two-tailed with α = 0.05. Averages are reported as median ± standard deviation. *N* denotes the number of independent animal samples, *n* the number of measurements per animal. When data from animals were pooled, we never used the measurement repetitions (*n*) taken from the individual animals, but a single averaged value for each animal. To determine whether there are acute effects of BPA and BPS, we used RM one-way ANOVAs. To determine whether there is an effect of 1-month exposure to BPA or BPS in comparison to the control group, we performed one-way ANOVAs and the Dunnett test for comparing each group with control. To determine whether there is an effect of one month exposure to EE2 in comparison to the control group, we performed unpaired *t* tests. Rate constants of exponential decay were compared using one-way ANOVA and Dunnett test. Differences in occurrence (in %) were compared using the Wilcoxon test with occurrence for control set as the hypothetical value. To test whether there are correlations between action potential amplitude and DP amplitude and between DP amplitudes, we used Spearman tests. To test whether data is distributed normally, we used the Shapiro–Wilk test.

### Reporting summary

Further information on research design is available in the Nature Research Reporting Summary linked to this article.

## Supplementary information

Supplemental Information

Description of Additional Supplementary Files

Supplementary Data 1

Supplementary Data 2

Supplementary Data 3

Reporting Summary

## Data Availability

The datasets generated and/or analyzed are available from the corresponding author on reasonable request. The source data for the graphs and charts in the main figures are present in the Supplementary data files.
