# Supplementary material for: Bisphenols exert detrimental effects on neuronal signaling in mature vertebrate brains
Source: Commun Biol. 2021 Apr 12;4:465. doi: 10.1038/s42003-021-01966-w (PMC8041872; doi:10.1038/s42003-021-01966-w)
Supplement: Supplementary file 1 — Supplemental Information [file 42003_2021_1966_MOESM1_ESM.pdf]

COMMUNICATIONS BIOLOGY

Supplementary Information for

Bisphenols exert detrimental effects on neuronal signaling in mature vertebrate brains

Elisabeth Schirmer, Stefan Schuster, Peter Machnik

Corresponding author: Peter Machnik
Email: peter.machnik@uni-bayreuth.de

This PDF file includes:

Supplementary Figure 1
Supplementary Table 1
Supplementary Table 2

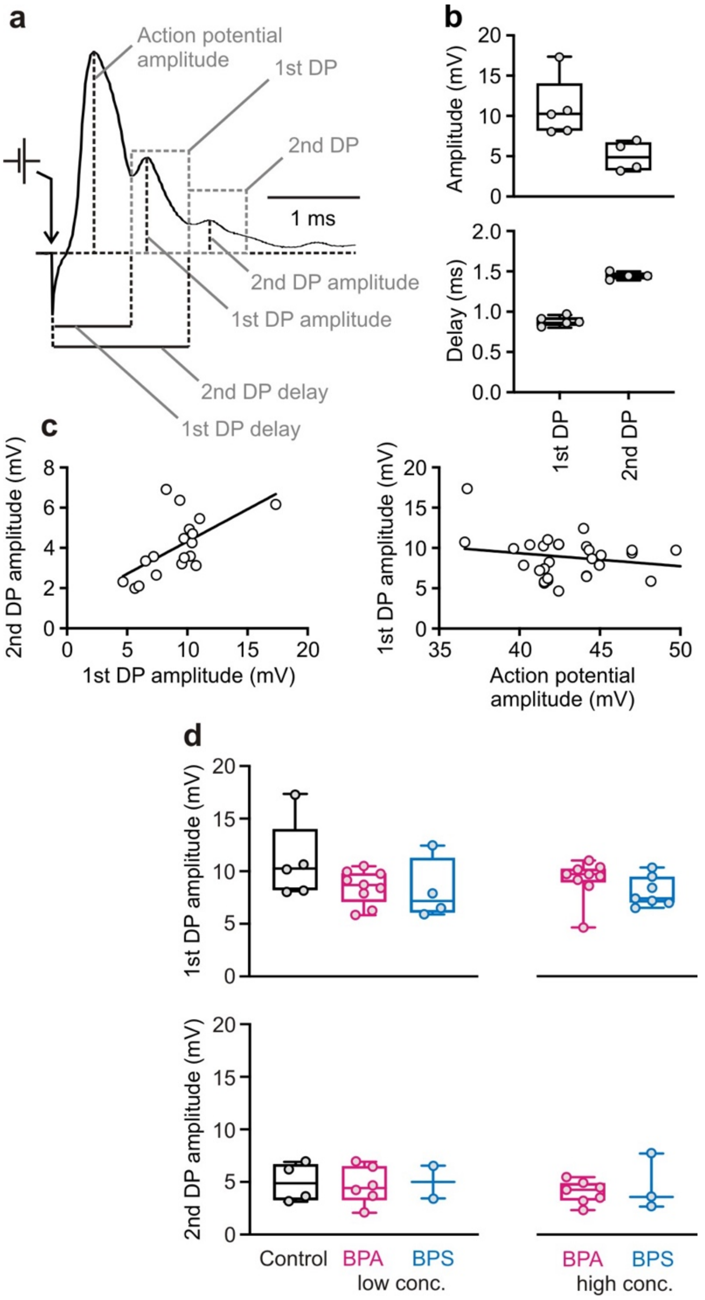

Supplementary Figure 1. Effects of BPA and BPS on neuronal backfiring.

(**a**) shows an exemplary action potential accompanied by delayed potentials (DPs) with illustration of how measurements were taken. (**b**) In controls, first DPs had on average an amplitude of 10.3 ± 3.8 mV (*N* = 5 independent samples; *n* = 9 to 31 measurements per fish) and followed 0.86 ± 0.06 ms after onset of the action potential. Second DPs had on average an amplitude of 4.9 ± 1.9 mV (*N* = 4 independent samples; *n* = 9 to 31) and followed 1.44 ± 0.05 ms after action potential onset. (**c**) The amplitude of the 2nd DP correlates with the amplitude of the first (*N* = 20 independent samples; 9 ≤ *n* ≤ 114; Spearman correlation: *P* = 0.01), the amplitude of the 1st DP and the amplitude of the action potential do not correlate (*N* = 33 independent samples; 9 ≤ *n* ≤ 114; Spearman correlation: *P* = 0.45). (**d**) Both BPA and BPS (in any concentration) did not affect the amplitude of the DPs (one-way ANOVA: $F \leq 1.54$; $R^2 \leq 0.18$; $P \geq 0.22$). Whiskers show the minimum and the maximum value, respectively.

Supplementary Table 1: Comparison of EE2 group and control

	Median ± SD		Unpaired t test	
	Control	EE2	t	P
Action potential				
Amplitude (mV)	41.4 ± 4.9	44.7 ± 2.7	2.453	**0.0240**
Delay (ms)	0.20 ± 0.03	0.15 ± 0.02	4.813	**0.0001**
Slope (V ms^{-1})	2.13 ± 0.33	0.82 ± 0.32	8.388	**<0.0001**
Area I_1 (mV*ms)	23.8 ± 3.1	23.1 ± 1.1	0.339	0.7384
	N = 13<br>9 ≤ *n* ≤ 31	*N* = 10<br>81 ≤ *n* ≤ 110		
Acoustically induced PSP				
Amplitude (mV)	7.1 ± 1.4	11.5 ± 2.5	5.128	**<0.0001**
Delay (ms)	7.71 ± 0.28	7.76 ± 0.30	0.437	0.6664
Slope (mV ms^{-1})	10.01 ± 2.66	10.76 ± 3.26	0.899	0.3787
Area I_1 (mV*ms)	117.5 ± 32.1	160.4 ± 38.4	2.543	**0.0189**
Area I_2 (mV*ms)	104.7 ± 20.2	111.6 ± 21.7	1.427	0.1683
	N = 13<br>8 ≤ *n* ≤ 29	*N* = 10<br>33 ≤ *n* ≤ 56		
Visually induced PSP				
Amplitude (mV)	10.4 ± 1.8	2.1 ± 1.4	10.22	**<0.0001**
Delay (ms)	30.0 ± 4.9	30.69 ± 5.3	0.276	0.7865
Slope (mV ms^{-1})	3.66 ± 0.82	2.52 ± 0.69	1.926	0.0710
Area I_1 (mV*ms)	398.0 ± 71.5	131.4 ± 47.6	10.33	**<0.0001**
Area I_2 (mV*ms)	357.1 ± 95.6	90.3 ± 68.0	7.173	**<0.0001**
	N = 8<br>7 ≤ *n* ≤ 21	*N* = 10<br>9 ≤ *n* ≤ 26		

Bold type highlights significant results. *N* denotes the number of independent animal samples, *n* the number of measurements per animal.

Supplementary Table 2: Data suggesting that the strong effects of BPA and BPS do not occur after short exposition of less than 1 hour.

BPA	Median ± SD			RM one-way ANOVA		
	0 µg L^{-1}	10 µg L^{-1}	1000 µg L^{-1}	F	R^2	P
Action potential						
Amplitude (mV)	43.9 ± 5.6	42.8 ± 3.8	42.6 ± 4.7	1.55	0.21	0.25
Delay (ms)	0.22 ± 0.03	0.21 ± 0.02	0.22 ± 0.02	1.16	0.16	0.35
Slope (V ms^{-1})	1.80 ± 0.49	1.81 ± 0.51	1.80 ± 0.55	1.35	0.18	0.30
Area I_1 (mV*ms)	21.7 ± 2.9	21.6 ± 2.2	21.8 ± 2.2	1.18	0.16	0.34
Acoustically induced PSP						
Amplitude (mV)	9.0 ± 2.5	9.1 ± 2.5	8.6 ± 2.6	3.35	0.40	0.09
Delay (ms)	7.50 ± 0.11	7.52 ± 0.12	7.49 ± 0.12	0.22	0.04	0.81
Slope (mV ms^{-1})	9.23 ± 2.55	8.53 ± 2.45	8.95 ± 2.17	3.59	0.42	0.07
Area I_1 (mV*ms)	132.4 ± 35.8	126.3 ± 35.9	125.7 ± 38.9	2.04	0.25	0.20
Area I_2 (mV*ms)	109.7 ± 26.1	102.8 ± 26.5	100.0 ± 30.8	3.30	0.35	0.11
Visually induced PSP						
Amplitude (mV)	8.5 ± 4.2	8.8 ± 2.9	7.1 ± 2.6	0.93	0.16	0.43
Delay (ms)	28.28 ± 4.59	29.24 ± 4.84	29.48 ± 4.47	2.90	0.33	0.09
Slope (mV ms^{-1})	2.85 ± 2.28	3.14 ± 2.06	2.95. ± 2.12	1.58	0.24	0.25
Area I_1 (mV*ms)	325.4 ± 73.1	312.0 ± 75.1	299.9 ± 66.7	2.37	0.28	0.14
Area I_2 (mV*ms)	190.8 ± 147.4	190.7 ± 98.4	189.0 ± 65.1	2.20	0.31	0.16
BPS	Median ± SD			RM one-way ANOVA		
	0 µg L^{-1}	10 µg L^{-1}	1000 µg L^{-1}	F	R^2	P
Action potential						
Amplitude (mV)	43.7 ± 5.4	43.3 ± 4.5	42.4 ± 4.5	0.57	0.10	0.58
Delay (ms)	0.20 ± 0.04	0.20 ± 0.04	0.20 ± 0.03	0.17	0.03	0.84
Slope (V ms^{-1})	1.95 ± 0.36	1.99 ± 0.29	1.99 ± 0.25	0.48	0.09	0.63
Area I_1 (mV*ms)	21.3 ± 3.2	21.0 ± 2.9	21.1 ± 2.5	0.13	0.02	0.88
Acoustically induced PSP						
Amplitude (mV)	9.6 ± 2.7	9.3 ± 2.8	9.7 ± 2.7	0.97	0.16	0.41
Delay (ms)	8.4 ± 1.6	7.9 ± 0.8	7.9 ± 0.4	0.73	0.16	0.48
Slope (mV ms^{-1})	9.40 ± 3.80	9.03 ± 3.13	9.33 ± 3.04	2.29	0.31	0.15
Area I_1 (mV*ms)	125.6 ± 44.2	122.3 ± 35.4	117.5 ± 31.8	0.49	0.09	0.62
Area I_2 (mV*ms)	108.5 ± 23.7	108.8 ± 19.2	112.7 ± 22.1	0.15	0.03	0.86
Visually induced PSP						
Amplitude (mV)	8.1 ± 2.6	9.3 ± 2.2	8.7 ± 2.5	0.03	0.01	0.96
Delay (ms)	27.84 ± 2.61	28.20 ± 2.47	28.26 ± 1.74	0.09	0.01	0.92
Slope (mV ms^{-1})	3.29 ± 0.65	3.32 ± 0.64	3.26 ± 0.61	1.57	0.28	0.27
Area I_1 (mV*ms)	319.1 ± 118.6	324.7 ± 104.5	330.1 ± 102.2	0.43	0.08	0.66
Area I_2 (mV*ms)	247.9 ± 140.2	255.7 ± 155.2	290.0 ± 160.1	2.36	0.37	0.16
