# Supplementary material for: Bisphenols exert detrimental effects on neuronal signaling in mature vertebrate brains
Source: Commun Biol. 2021 Apr 12;4:465. doi: 10.1038/s42003-021-01966-w (PMC8041872; doi:10.1038/s42003-021-01966-w)
Supplement: Supplementary file 2 — Description of Additional Supplementary Files [file 42003_2021_1966_MOESM2_ESM.pdf]

Description of Additional Supplementary Files

File Name: Supplementary Data 1

Description: Supplemental Data to Figure 2

File Name: Supplementary Data 2

Description: Supplemental Data to Figure 3

File Name: Supplementary Data 3

Description: Supplemental Data to Figure 4
