# Supplementary material for: Bisphenols exert detrimental effects on neuronal signaling in mature vertebrate brains
Source: Commun Biol. 2021 Apr 12;4:465. doi: 10.1038/s42003-021-01966-w (PMC8041872; doi:10.1038/s42003-021-01966-w)
Supplement: Supplementary file 6 — Reporting Summary [file 42003_2021_1966_MOESM6_ESM.pdf]

nature research

Corresponding author(s): Peter Machnik

Last updated by author(s): Feb 23, 2021

Reporting Summary

Nature Research wishes to improve the reproducibility of the work that we publish. This form provides structure for consistency and transparency in reporting. For further information on Nature Research policies, see our Editorial Policies and the Editorial Policy Checklist.

Statistics

For all statistical analyses, confirm that the following items are present in the figure legend, table legend, main text, or Methods section.

n/a	Confirmed	
[ ]	[x]	The exact sample size (*n*) for each experimental group/condition, given as a discrete number and unit of measurement
[ ]	[x]	A statement on whether measurements were taken from distinct samples or whether the same sample was measured repeatedly
[ ]	[x]	The statistical test(s) used AND whether they are one- or two-sided<br>*Only common tests should be described solely by name; describe more complex techniques in the Methods section.*
[x]	[ ]	A description of all covariates tested
[ ]	[x]	A description of any assumptions or corrections, such as tests of normality and adjustment for multiple comparisons
[ ]	[x]	A full description of the statistical parameters including central tendency (e.g. means) or other basic estimates (e.g. regression coefficient) AND variation (e.g. standard deviation) or associated estimates of uncertainty (e.g. confidence intervals)
[ ]	[x]	For null hypothesis testing, the test statistic (e.g. *F*, *t*, *r*) with confidence intervals, effect sizes, degrees of freedom and *P* value noted<br>*Give P values as exact values whenever suitable.*
[x]	[ ]	For Bayesian analysis, information on the choice of priors and Markov chain Monte Carlo settings
[x]	[ ]	For hierarchical and complex designs, identification of the appropriate level for tests and full reporting of outcomes
[x]	[ ]	Estimates of effect sizes (e.g. Cohen's *d*, Pearson's *r*), indicating how they were calculated

Our web collection on statistics for biologists contains articles on many of the points above.

Software and code

Policy information about availability of computer code

Data collection	We used Spike2 (version 6; Cambridge Electronic Design Limited, Cambridge, UK) for collecting data
Data analysis	We used GraphPad Prism 8.2.1 (GraphPad Software, Inc., La Jolla, CA, USA) for statistical analyses

For manuscripts utilizing custom algorithms or software that are central to the research but not yet described in published literature, software must be made available to editors and reviewers. We strongly encourage code deposition in a community repository (e.g. GitHub). See the Nature Research guidelines for submitting code & software for further information.

Data

Policy information about availability of data

All manuscripts must include a data availability statement. This statement should provide the following information, where applicable:

- Accession codes, unique identifiers, or web links for publicly available datasets
- A list of figures that have associated raw data
- A description of any restrictions on data availability

The data sets generated during and/or analysed during the current study are available from the corresponding author on reasonable request.

Field-specific reporting

Life sciences study design

All studies must disclose on these points even when the disclosure is negative.

Sample size	Since it was not known whether there is an effect of BPA, BPS or EE2 on neurons in the adult brain at all or what the strength of the effect is, we chose a group size that had been shown in a previous study (Machnik, Schirmer & Schuster (2018; Sci. Rep. 8, DOI:10.1038/ s41598-018-36130-8)) to be suitable to reliably detect even small effects on the Mauthner neuron.
Data exclusions	No data were excluded from analyses.
Replication	All attempts of replication were successful. Effects of BPA, BPS and EE2 on neuronal function and processsing of sensory information were similar in all exposed experimental animals. Data collected in different experimental animals were independent from each other.
Randomization	The experimental animals were always allocated randomly to the experimental groups.
Blinding	Investigators were blinded to group allocation during data collection and data analysis.

Reporting for specific materials, systems and methods

We require information from authors about some types of materials, experimental systems and methods used in many studies. Here, indicate whether each material, system or method listed is relevant to your study. If you are not sure if a list item applies to your research, read the appropriate section before selecting a response.

Materials & experimental systems

n/a	Involved in the study	
x		Antibodies
x		Eukaryotic cell lines
x		Palaeontology and archaeology
	x	Animals and other organisms
x		Human research participants
x		Clinical data
x		Dual use research of concern

Methods

n/a	Involved in the study	
x		ChIP-seq
x		Flow cytometry
x		MRI-based neuroimaging

Animals and other organisms

Policy information about studies involving animals; ARRIVE guidelines recommended for reporting animal research

Laboratory animals	We used N = 98 adult goldfish (Carassius auratus, Cypriniformes) of either sex with an average standard length of 69.5 ± 7.8 mm (range from 56.5 to 100 mm) and an average weight of 10.3 ± 3.8 g (range from 6.7 to 20.8 g). The information is added in Methods.
Wild animals	The study did not involve wild animals.
Field-collected samples	The study did not involve samples collected from the field.
Ethics oversight	Animal care procedures, surgical procedures and experimental procedures were in accordance with all relevant guidelines and regulations of the German animal protection law and explicitly approved by state councils (Regierung von Unterfranken, Würzburg, Germany). The respective information is added in Methods.

Note that full information on the approval of the study protocol must also be provided in the manuscript.
